# Impact of Scotland’s Comprehensive, Smoke-Free Legislation on Stroke

**DOI:** 10.1371/journal.pone.0062597

**Published:** 2013-05-08

**Authors:** Daniel F. Mackay, Sally Haw, David E. Newby, Peter Langhorne, Suzanne M. Lloyd, Alex McConnachie, Jill P. Pell

**Affiliations:** 1 Institute of Health and Wellbeing, University of Glasgow, Glasgow, United Kingdom; 2 School of Nursing, Midwifery and Health, University of Stirling, Stirling, United Kingdom; 3 Centre for Cardiovascular Science, University of Edinburgh, Edinburgh, United Kingdom; 4 Institute of Cardiovascular and Medical Sciences, University of Glasgow, Glasgow, United Kingdom; Fundación para la Prevención y el Control de las Enfermedades Crónicas No Transmisibles en América Latina (FunPRECAL), Argentina

## Abstract

**Background:**

Previous studies have reported a reduction in acute coronary events following smoke-free legislation. Evidence is lacking on whether stroke is also reduced. The aim was to determine whether the incidence of stroke, overall and by sub-type, fell following introduction of smoke-free legislation across Scotland on 26 March 2006.

**Methods and Findings:**

A negative binomial regression model was used to determine whether the introduction of smoke-free legislation resulted in a step and/or slope change in stroke incidence. The model was adjusted for age-group, sex, socioeconomic deprivation quintile, urban/rural residence and month. Interaction tests were also performed. Routine hospital administrative data and death certificates were used to identify all hospital admissions and pre-hospital deaths due to stroke (ICD10 codes I61, I63 and I64) in Scotland between 2000 and 2010 inclusive. Prior to the legislation, rates of all stroke, intracerebral haemorrhage and unspecified stroke were decreasing, whilst cerebral infarction was increasing at 0.97% per annum. Following the legislation, there was a dramatic fall in cerebral infarctions that persisted for around 20 months. No visible effect was observed for other types of stroke. The model confirmed an 8.90% (95% CI 4.85, 12.77, p<0.001) stepwise reduction in cerebral infarction at the time the legislation was implemented, after adjustment for potential confounders.

**Conclusions:**

Following introduction of national, comprehensive smoke-free legislation there was a selective reduction in cerebral infarction that was not apparent in other types of stroke.

## Introduction

In Scotland, the Smoking, Health and Social Care (Scotland) Bill prohibited smoking in virtually all wholly and substantially enclosed public places and workplaces from 26 March 2006, including bars, restaurants, cafes and private members clubs. The few exemptions included designated rooms in residential accommodation, adult care homes, hotels and psychiatric units. The law is enforced by Environmental Health Officers. It became illegal both to smoke and to permit smoking in smoke-free premises. Both the individuals who flout the legislation and the managers of premises in which they are permitted to do so face fixed penalties. Refusal or failure to pay either may result in prosecution and an increased fine. The primary aim of the legislation was to protect non-smokers from the adverse health effects of exposure to environmental tobacco smoke (ETS), but it was anticipated that health benefits might also accrue from a reduction in tobacco consumption and smoking prevalence in the general population. No other new, national tobacco control measures were introduced over the study period.

Smoking is one of the major risk factors for stroke [1]. Cerebral blood flow is reduced in chronic smokers [2], due to an increase in platelet adhesiveness [3], fibringogen levels [4], blood viscosity [4], and vascular resistance [5]. Compared with non-smokers, active smoking is associated with a 2–4 fold increased risk of stroke [6]. In comparison with non-smokers protected from environmental tobacco smoke, the risk is up to 6-fold [7]. Smoking is also associated with poorer functional outcome among those who suffer stroke [8]. The increased risk of stroke among smokers persists for several years after smoking cessation [4], suggesting that in addition to pro-thrombotic effects, cigarette smoke predisposes to atherogenesis. Active smoking is associated with both ischaemic and haemorrhagic stroke [6], [9], but the relative risk may be higher for ischaemic strokes, particularly those due to thromboembolic infarction [10]. Active smoking is not associated with atrial fibrillation [11] and, therefore, is not associated with ischaemic stroke due to cardiac emboli [10].

ETS is a mixture of non-inhaled side-stream smoke from burning cigarette tips and main-stream smoke exhaled by smokers. Many toxic gases and small, respirable particles are present in higher concentrations in ETS than in the mainstream smoke inhaled by smokers. Exposure to small amounts of ETS significantly and rapidly increases platelet aggregation, thrombosis, endothelial dysfunction, and inflammation [12]–[16], resulting in levels of homocysteine, C-reactive protein, fibrinogen and oxidized low density lipoprotein cholesterol of similar magnitude to those in active smokers [17]. An association between ETS exposure and risk of all strokes has now been demonstrated in case-control [7], cross-sectional [18], and cohort studies [19], [20]. A number of studies have focused on ischaemic stroke [10], [19], [21], [22]. In a case-control study, You et al. demonstrated an association between a spouse smoking and risk of ischaemic stroke among both active smokers and non-smokers [21]. There is evidence of a dose response in relation to the number of cigarettes smoked by a spouse [10], [18], [21], [22], the amount of time spent smoking [21], and the number of years they have smoked [18]. In a cross-sectional study, He et al. demonstrated a significant association between ETS and both total and ischaemic stroke (OR 1.56, 95% CI 1.03–2.35), but not haemorrhagic stroke (OR 1.10, 95% CI 0.52–2.34) (22). In a case-control study of sub-arachnoid haemorrhage, Anderson et al demonstrated a strong association with active smoking but no association with exposure to ETS [23]. Most studies have relied on self-reported ETS exposure. In the British Regional Heart Study, ETS was measured via cotinine assay [24]. The investigators demonstrated a dose relationship between cotinine level and risk of stroke but this did not reach statistical significance.

Many studies have demonstrated a significant reduction in the incidence of acute coronary events following introduction of smoke-free legislation [25], [26]. Smoking increases the risk of acute coronary events through a prothrombotic effect. Therefore, we hypothesised that smoke-free legislation may also have reduced the risk of stroke overall, and thrombotic stroke specifically.

## Methods

### Data Sources and Exclusion Criteria

Scotland has a population of around 5.1 million. The Scottish Morbidity Record (SMR01) collects information on all admissions to acute hospitals in Scotland including admission date, urgency of admission, age, sex and postcode of residence. It also collects the principal and secondary disease codes using the International Classification of Diseases (ICD). The General Register Office for Scotland collates death certificate data on all deaths that occur in Scotland including those that occur in the community, as well as in-hospital. The death certificate data include date of death, age, sex, postcode of residence and the primary underlying cause of death, classified using ICD. The individual-level data from both systems are linked by the Information and Statistics Division (ISD) of National Health Services Scotland enabling recurrent events in the same individual to be identified. We used these data to identify all emergency admissions and pre-hospital deaths due to a principal diagnosis of stroke that occurred in Scotland between 2000 and 2010 inclusive. We excluded from our analysis people who had suffered a stroke during the previous ten years. Since this was a secondary analysis of existing, anonymised data we did not obtain individual participant consent. Permission to use both sets of data was granted by the Privacy Advisory Committee of the Information and Statistics Division.

### Definitions

Cerebral infarction was defined as a principal diagnosis of ICD10 I63, intracerebral haemorrhagic stroke as a principal diagnosis of ICD10 I61, and unspecified stroke as a principal diagnosis of ICD10 I64. Confirmed strokes included both cerebral infarction and intra-cerebral haemorrhage and excluded unspecified stroke, and all strokes included both confirmed and unspecified stroke. Events included both pre-hospital deaths and hospital admissions, irrespective of whether the patient was discharged alive or died in-hospital. Postcodes of residence were aggregated into datazones of residence (http://www.scotland.gov.uk/Topics/Statistics/SIMD/Overview). There are 6,505 datazones in Scotland with a median population of 769. Information on 38 indicators across 7 domains, namely: income, employment, health, education, skills and training, housing, geographic access and crime, are collected at the Census and used to attribute a Scottish Index of Multiple Deprivation (SIMD) to each datazone. The Scottish Indices of Multiple Deprivation are then aggregated into population quintiles of deprivation ranging from 1 (most affluent) to 5 (most deprived). Categorisation of postcode of residence into urban and rural was based on the Scottish Executive’s Classification system (http://www.scotland.gov.uk/Resource/Doc/47251/0028898.pdf). Large urban areas and other urban areas were both coded as urban and the remainder were coded as rural.

### Statistical Analyses

We determined the frequency of stroke events each month for sub-groups defined by age (<60 and ≥60 years), sex, deprivation quintile and urban/rural classification. Annual population counts in each of these sub-groups, for each month, were estimated by linear extrapolation of annual small area population estimates from the General Registrar (Scotland) Office (http://www.gro-scotland.gov.uk/statistics/theme/population/estimates/special-area/sape/archive/index.html) for data zones obtained from the Scottish 2001 Census to which were matched the urban-rural indicator and datazone quintile of deprivation. Adjustments to the population counts were made for leap years. Separate negative binomial regression models were fitted for all strokes and sub-types of stroke. The models allowed for an underlying trend in incidence rates throughout the whole study period, and for a step change and change in trend after implementation of the smoke-free legislation. The models were adjusted for actual age group, sex, deprivation quintile, urban/rural classification, and month and year. We included an offset term to account for different population sizes and the number of days in each month. The negative binomial models were compared to Poisson equivalents using the Akaike information criterion (AIC) and Bayesian information criterion (BIC) statistics and these confirmed that negative binomial regression was more appropriate than Poisson regression for our data. There was no evidence of over-dispersion within the models. The models were extended to include interaction terms to test for differences in the post-legislation change in incidence trend between sub-groups of the population (after allowing for different pre-legislation trends); specifically age group, sex, deprivation quintile and urban/rural classification.

## Results

Over the eleven year period, between 2000 and 2010 inclusive, there were 86,835 stroke events: 35,810 cerebral infarctions, 9,210 intra-cerebral haemorrhages, and 41,815 unspecified strokes.

**Figure 1:**
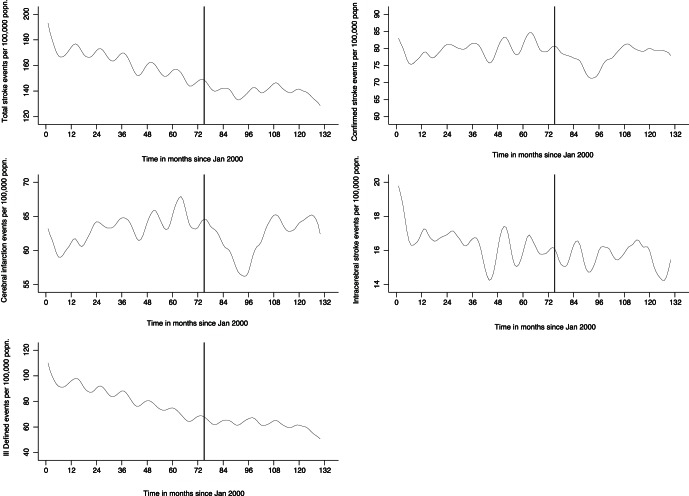
Time trends in stroke incidence (hospital admissions plus pre-hospital deaths). Footnote: graphs show lowess smoothed incidence rates with a bandwidth of 0.1.

Of those 86,835, complete data was available on 85,662 (98.6%): 35,308 cerebral infarctions, 9,050 intra-cerebral haemorrhages and 41,304 unspecified strokes. Table 1 shows the breakdown by age, sex, residence and deprivation. On visual inspection, all stroke, intracerebral haemorrhage and unspecified stroke were decreasing in incidence prior to implementation of the legislation (Figure 1 & Figure S1). In contrast, the incidence of cerebral infarction was rising (Figure 1 & Figure S1). Following implementation of the legislation there appeared to be a dramatic fall in cerebral infarction incidence that persisted for around 20 months before partially reverting to pre-legislation levels (Figure 1 & Figure S1). Visual inspection of the graphs for the other types of stroke suggested no obvious effect of the legislation (Figure 1 & Figure S1).

**Table 1:** Absolute changes in cerebral infarction incidence referent to pre-legislation slope.

			Step change on 26 March 2006		Change in slope post 26 March 2006	
		n	% (95% CI)	P value	% (95% CI)	P value
**Overall** *		35,308	−8.90 (−12.77, −4.85)	<0.001	−0.40 (−1.86, 1.07)	0.590
**Sex**	**male**	17,855	−9.49 (−15.40, −3.17)	0.004	0.06 (−2.24, 2.43)	0.957
	**female**	17,453	−8.32 (−12.99, −3.39)	0.001	−0.85 (−2.60, 0.93)	0.347
**Age (years)**	**<60**	5,652	−9.26 (−13.50, −4.82)	<0.001	0.19 (−1.46, 1.87)	0.821
	**≥60**	29,656	−7.42 (−15.65, 1.61)	0.105	−3.32 (−5.45, −1.15)	0.003
**Residence**	**urban**	25,233	−8.68 (−13.14, −3.99)	<0.001	−0.59 (−2.45, 1.31)	0.540
	**rural**	10,075	−9.39 (−16.64, −1.50)	0.021	0.11 (−1.97, 2.23)	0.919
**Deprivation**	**1 affluent**	5,171	−10.88 (−16.41, −4.98)	<0.001	−2.01 (−3.50, −0.54)	0.007
**quintile**	**2**	5,950	−6.92 (−10.33, −3.37)	<0.001	1.30 (0.15, 2.46)	0.027
	**3**	6,790	−13.29 (−24.87, 0.07)	0.051	−2.93 (−6.05, 0.30)	0.181
	**4**	8,212	−1.73 (−12.56, 10.43)	0.769	0.41 (−1.91, 2.77)	0.733
	**5 deprived**	9,185	−10.31 (−14.81, −5.59)	<0.001	2.42 (−3.00, 8.13)	0.389

CI confidence interval.

* adjusted for sex, age, residence and deprivation quintile.

In the statistical model, the incidence of cerebral infarction increased at 0.97% per annum prior to the legislation, after adjusting for changes in age, sex, deprivation quintile and urban/rural residence, as well as month of event. At the time of implementation there was a stepwise absolute reduction of 8.90% (95% CI 4.85, 12.77, p<0.001) (Table 1). The stepwise reduction in incidence reached statistical significance in most sub-groups of the population (Table 1). None of the formal tests for interactions between sub-groups were statistically significant.

The incidence of confirmed stroke increased at 0.35% per annum prior to the legislation, after adjusting for changes in age, sex, deprivation quintile and urban/rural residence, as well as month. At the time of implementation there was a stepwise absolute reduction of 6.65% (95% CI 2.95, 10.22, p<0.001) (Table 2). The stepwise reduction reached statistical significance in most sub-groups of the population (Table 2). None of the formal tests for interactions between sub-groups were statistically significant.

**Table 2:** Absolute changes in confirmed stroke (cerebral infarction plus intracerebral haemorrhage) incidence referent to pre-legislation slope.

			Step change on 26 March 2006		Change in slope post 26 March 2006	
		n	% (95% CI)	P value	% (95% CI)	P value
**Overall** *		44,358	−6.65 (−10.22, −2.95)	<0.001	−0.23 (−1.49, 1.06)	0.728
**Sex**	**male**	22,531	−6.81 (−11.82, −1.57)	0.012	−0.12 (−2.11, 1.92)	0.917
	**female**	21,827	−6.51 (−11.31, −1.45)	0.012	−0.32 (−1.91, 1.29)	0.227
**Age (years)**	**<60**	7,536	−7.13 (−10.87, −3.24)	<0.001	0.36 (−1.08, 1.81)	0.628
	**≥60**	36,822	−4.76 (−13.84, 5.29)	0.341	−2.77 (−4.73, −0.78)	0.007
**Residence**	**urban**	31,653	−6.12 (−10.02, −2.06)	0.003	−0.35 (−1.99, 1.31)	0.673
	**rural**	12,705	−7.87 (−14.99, −0.15)	0.046	0.14 (−1.61, 1.92)	0.879
**Deprivation**	**1 affluent**	6,673	−9.68 (−14.61, −4.47)	<0.001	−1.78 (−3.81, 0.28)	0.090
**quintile**	**2**	7,523	−4.63 (−8.09, −1.03)	0.012	0.91 (0.02, 1.81)	0.045
	**3**	8,608	−11.82 (−22.56, 0.41)	0.058	−1.89 (−5.00, 1.33)	0.246
	**4**	10,252	−2.20 (−9.44, 5.62)	0.571	0.89 (−1.71, 3.56)	0.506
	**5 deprived**	11,302	−2.47 (−5.55, 0.71)	0.127	1.67 (−2.44, 5.95)	0.432

CI confidence interval.

* adjusted for sex, age, residence and deprivation quintile.

## Discussion

Following introduction of national smoke-free legislation, there was a significant stepwise reduction in the incidence of cerebral infarction relative to pre-legislation levels. By contrast, there was no significant impact on intra-cerebral haemorrhage or unspecified stroke. The reduction in cerebral infarction lasted for around 20 months before partially reverting to pre-legislation levels.

Following implementation of Scotland’s smoke-free legislation in March 2006, there has been a very high level of compliance [27], and a reduction in overall exposure to ETS in the general population [28]. There was also a dramatic increase in smoking quit attempts at the beginning of 2006, in anticipation of the legislation, resulting in a significant fall in smoking prevalence [29]. However, the beneficial effects on active smoking were not maintained over time and, one year following the legislation, both had reverted to levels not significantly different from the underlying trends [29]. Whilst smoke free legislation may encourage reduced consumption and increased quit attempts, additional measures appear to be required if benefits are to be maintained in the long-term. In the absence of information on the smoking status of stroke patients, we can only speculate as to the relative contribution of reduced active smoking and ETS to the fall in cerebral infarctions at the time that smoke-free legislation was implemented. After 20 months the reduction was partially reversed but rates remained lower than predicted from the pre-legislation trends. It is plausible that the partial reversal was due to the failure to maintain a reduction in smoking prevalence.

In comparison with coronary heart disease, there have been relatively few studies that have examined the impact of smoke-free legislation on cerebrovascular disease. A study conducted in Ontario, Canada, included stroke in the definition of cardiovascular disease (along with acute myocardial infarction and angina) and demonstrated a reduction following smoke-free legislation [30]. To date, there have been three published studies that have examined the impact of smoke-free legislation on stroke specifically, and none have reported the effect on sub-types of stroke. Following state-wide legislation in Arizona, there were statistically significant reductions in admissions for stroke in those counties which did not have pre-existing local restrictions [31]. The introduction of comprehensive legislation across New York State produced no significant reduction in total admissions for all-cause stroke over and above those following previous partial and local restrictions [32]. A study conducted in New Zealand applied a wide definition of stroke, including sub-dural and extra-dural haemorrhage, as well as intra-cerebral haemorrhages and cerebral infarction [33]. The investigators observed a significant fall in the crude number of admissions in the year following legislation but this did not remain statistically significant following adjustment for potential confounders.

In our study, we had access to high-quality data covering the whole of Scotland. Therefore, the results cannot be attributed to changes in hospital catchment areas or referral practice. We had access to data on pre-hospital deaths, enabling us to examine overall incidence rather than hospital admissions. Therefore, any post-legislation changes cannot be attributed to a change in the natural history or pre-hospital management of the condition. Because we used record linkage to identify recurrent events in the same individual we were able to exclude people with a past history of stroke and only include incident events. This enabled us to separate the association between legislation and incidence, separately from any association with prognosis. In our study, we were able to test for interactions with age, sex, deprivation and urban/rural classification.

Our assumptions regarding the underlying trend were based on data from six years prior to the legislation. Inclusion of more years would have required use of ICD9 as well as ICD10 codes and could have led to artefactual changes over time due to non-exact mapping. Exploratory analyses (not shown) using data prior to 2000 showed evidence of a step changes in event rate at this time, suggesting this to be the case. Because we used routine data sources, we did not have access to information on smoking status or to either self-reported or biochemical measures of level of ETS exposure pre- and post-legislation. Therefore, we were unable to determine whether the observed decline in cerebral infarction occurred in non-smokers, smokers or both, and we cannot surmise whether the association was mediated via a reduction in ETS exposure or reduced consumption and increased quitting among smokers. Scottish routine data undergo regular quality assurance checks. However, as with any retrospective, secondary analyses some data may be inaccurate or incomplete. From audit data, we know that the proportion of patients admitted with stroke who have undergone scans has increased over the study period, resulting in an underlying decline in unspecified stroke and increase in stroke classified as cerebral infarction [34]. However, changes in scanning rates were minor in the two years before and after the smoking legislation was introduced [34]. Over time there have been many changes in stroke management, such as an increase in the percentage of stroke patients admitted to hospital, the introduction of multidisciplinary stroke teams and units, educational campaigns to raise public awareness of stroke and the need to phone for an ambulance, and use of thrombolysis, which have impacted on community and in-hospital case-fatality. Therefore, it was not possible to analyse fatal and non-fatal strokes separately and determine the specific effect of the legislation. There were no new, national tobacco control initiatives, other than the smokefree legislation, introduced over the period studied. However, in any natural history experiment it is impossible to identify and exclude the effects of all the interventions and influences that may have occurred.

### Conclusions

Our study is the first to demonstrate a reduction in cerebral infarction incidence following comprehensive, smoke-free legislation. Additional studies are required to corroborate our findings in other countries, and to explore whether the reduction is due to reduced risk in smokers or protection from ETS.

## Supporting Information

Figure S1 Time trends in stroke incidence (hospital admissions plus pre-hospital deaths) (equivalent to Figure 1 (confirmed stroke) and figure 1 (cerebral infarction) with the addition of a regression line and 95% confidence intervals).
(EPS)
